# Optimizing Positive Airway Pressure Compliance and Outcomes in Rural Patients with Obstructive Sleep Apnea Through Telehealth

**DOI:** 10.3390/ijerph22040522

**Published:** 2025-03-29

**Authors:** Jenna R. Cooper, Kristi A. Acker, James D. Geyer, Monica M. Henderson, Randi Henderson-Mitchell, John C. Higginbotham

**Affiliations:** 1 Alabama Neurology and Sleep Medicine, Tuscaloosa, AL 35406, USA; jdgeyer@ua.edu (J.D.G.); mhenderson@nctpc.com (M.M.H.); 2 School of Nursing, The University of Alabama, Tuscaloosa, AL 35401, USA; kaacker@ua.edu; 3 Institute for Rural Health Research, College of Community Health Sciences, The University of Alabama, Tuscaloosa, AL 35401, USA; rjhenderson@ua.edu; 4 Research Institute of Pharmaceutical Sciences, The University of Mississippi, Oxford, MS 38677, USA; jch@olemiss.edu

**Keywords:** obstructive sleep apnea (OSA), positive airway pressure (PAP) compliance, rural setting, telehealth, sleep medicine, telemedicine, health education programs, rural health networks

## Abstract

Introduction: Telehealth approaches have demonstrated benefits in improving positive airway pressure (PAP) compliance in patients with obstructive sleep apnea (OSA), improving access to healthcare resources, and improving health outcomes for rural communities. Methods: This quality improvement (QI) pilot study implemented weekly telemedicine visits for four weeks of PAP therapy in rural patients newly diagnosed with OSA. Epworth Sleepiness Scale (ESS) scores were compared prior to and at one month of therapy. PAP compliance was compared between rural patients who received the telemedicine intervention and a group of patients not receiving the telemedicine intervention. Results: Compliance rates were higher in the intervention group. There was not a significant difference in compliance for the intervention group (M = 63.22, SD = 32.78) versus the control group (M = 46.40, SD = 36.24), t (46) = 1.69, *p* = 0.099. ESS scores were significantly greater prior to one month of therapy (M = 8.38, SD = 5.70) compared to after one month of therapy (M = 2.83, SD = 2.65), t (23) = 5.22, *p* < 0.001, d = 1.07. Discussion: This QI pilot study utilized telemedicine to remove barriers to care, improve PAP compliance, and improve health outcomes for this underserved, rural population.

## 1. Introduction

Obstructive sleep apnea (OSA) is a common and treatable sleep-related breathing disorder associated with significant morbidity and economic cost. OSA is characterized by intermittent partial or complete airway obstruction or pauses in breathing. OSA with its associated reduction or interruption of airflow during sleep, can result in hypercapnia, hypoxemia, fluctuations in blood pressure, and fragmented sleep. Clinical symptoms include snoring, gasping, choking, morning headache, nocturnal urination, dry mouth upon waking, and daytime sleepiness [1]. Untreated sleep apnea may contribute to the risk of stroke, coronary artery disease, arrhythmias such as atrial fibrillation, hypertension, and increased cardiovascular mortality. Multiorgan damage makes OSA an economic burden for patients, families, and society. Positive airway pressure (PAP) is the optimal treatment for OSA. Lack of compliance with PAP therapy may cause complications that affect patients’ quality of life and health. People living in rural areas often have limited access to healthcare as well as barriers to care which can limit treatment of OSA. Healthy People 2030 has included a goal to increase access to comprehensive, high-quality healthcare services. Telehealth improves access to care for people living in rural, underserved areas.

Patient education and counseling are extremely important for creating patient–provider rapport, improving PAP compliance, and enhancing satisfaction with care. In-person PAP management is the typical approach, but telehealth options are increasingly common. For rural and underserved regions, telehealth extends the reach of sleep providers and overcomes some barriers to care experienced by these populations. Telehealth solutions need not surpass in-person interactions. In this case, telehealth protocols must provide appropriate training, at least equal compliance, and improve patient satisfaction.

Untreated sleep apnea can impair concentration, decision-making, memory, and executive function [2]. Excessive daytime sleepiness from untreated sleep apnea can also result in danger and increased accidents while driving. Healthy People 2030 has included an objective to decrease the rate of motor vehicle crashes due to drowsy driving. The excessive sleepiness associated with untreated or inadequately treated OSA increases the risk of injury and absenteeism, further increasing the burden on families and society.

The American Academy of Sleep Medicine (AASM) commissioned a white paper to assess the financial and health impact of untreated sleep apnea. The research found that approximately 12% or 29.4 million adults living in the United States have OSA, with nearly 80% of the population undiagnosed. They estimated that undiagnosed OSA cost the United States approximately $149.6 billion dollars in 2015 [3]. The estimated costs include $86.9 billion in lost productivity, $26.2 billion in motor vehicle accidents, and $65 billion in workplace accidents. Sleep apnea also costs $30 billion annually in increased healthcare utilization and medication costs related to worsening of comorbidities including hypertension, heart failure, diabetes, and depression. OSA is also associated with cardiac arrythmia and stroke [4]. The impact of insufficiently treated OSA is even less well recognized.

The National Sleep Foundation reports that approximately 25% of men and 10% of women suffer from obstructive sleep apnea. While increasing age and weight increase the risk of OSA, it can affect people of any age, including children [5]. Minority and multiple minority patients are at higher risk of OSA and complications from lack of treatment. African American children are twice as likely to have OSA [6].

Geography significantly impacts access to health care. Healthy People 2030 has designated Health Care Access and Quality to be one of the five domains of the social determinants of health. Social determinants of health describe where people are born and live and affect quality of life and health [7]. Rural settings often lack access to hospitals and general healthcare resources. The USDA defines a rural area as an open countryside with less than five hundred people per square mile, places with fewer than 2500 people per square mile, and places with fewer than 2500 people. Nearly sixty million Americans live in rural areas [8]. These same groups face a variety of additional barriers to care.

Untreated sleep apnea inflicts a large global health, financial, and safety burden. With almost one billion people affected worldwide, several barriers to care limit effective treatment [9]. Individuals travelling farther for sleep care are more likely to report symptoms of OSA despite not having a formal diagnosis and have more severe apnea upon diagnosis. Therefore, many rural patients with significant but less obvious OSA go undiagnosed. Remote clinics and telehealth have demonstrated improved therapy compliance in newly diagnosed sleep apnea [9]. A meta-analysis of eleven randomized controlled trials found telehealth interventions beneficial for PAP compliance in patients with OSA [10]. Telehealth has the potential to offer more convenient care and reduce travel [11]. Telehealth can include telecommunications technology such as telephones, wireless modems, computer-based telecommunication systems, and smartphone applications to provide health service information remotely and achieve remote communication between patients and medical staff [10]. Enhancing both the platform and content delivered are vital for optimized patient outcomes.

### 1.1. Problem Statement

OSA is a common and treatable condition associated with significant morbidity and economic cost [6]. People living in rural areas have geographical barriers to healthcare since they must travel farther to clinics and hospitals and often have fewer specialty services offered in these areas. These same patients often face multiple additional barriers to care including financial and lack of social support. Healthy People 2030 includes initiatives to increase the number of people screened and treated for OSA and has also designated Health Care Access and Quality to be one of the five domains of the social determinants of health. Telehealth solutions can help mitigate barriers to care and have demonstrated benefit in improving positive PAP therapy compliance in a cost-effective manner in limited populations [12].

Many articles discuss the negative health impacts of untreated sleep apnea, but few have addressed the overwhelming barriers faced by patients living in rural areas. We developed a Sleep Health Optimization Program (SHOP) to mitigate the significant health and societal costs of OSA by improving PAP compliance through utilization of weekly telehealth visits for the first month of PAP therapy in rural patients newly diagnosed with OSA. SHOP prioritizes sleep health by improving access to care and subsequently improving PAP compliance, overall health, and level of daytime sleepiness for this underserved rural population.

### 1.2. Organizational Gap Analysis of Project Site

A theory-practice gap occurs when evidence-based practice is not integrated into clinical practice [13]. Standards of care and practice guidelines have been established by the American Academy of Sleep Medicine (AASM) and are available to all sleep specialists. Unfortunately, physicians and sleep programs do not consistently adhere to the recommendations regarding patient support and monitoring during the initial period of PAP therapy, thereby creating a theory–practice gap. Compliance with treatment is a key factor in improving the health status of patients diagnosed with OSA. Furthermore, enhancing patient satisfaction and patient–provider communication are vitally important aspects of patient care and compliance.

### 1.3. Current State

Currently, our practice operates a rural clinic, an urban clinic, and credentialed sleep centers at both sites. An industry standard for definition of compliance with PAP therapy is using the PAP machine for at least four hours per night, for one thirty-day period, during a ninety-day compliance window. Many insurers require PAP usage to be monitored during the first ninety days of therapy or after receiving a new machine. The no-show rate for visits and failed compliance for patients living in rural areas is substantially higher than that of patients living in urban areas at both practice sites (internal quality assurance program findings, 18 January 2023). Multiple barriers to care contribute to this problem, including lack or cost of transportation, educational background, perceived need for treatment, and most importantly, decreased access to healthcare including specialty services. We initiated a sleep medicine telehealth support program in our practice during the COVID pandemic and we continue to offer these telehealth services since they were so well received by patients, especially those living in rural areas.

The estimated population of Alabama is 4,893,186 with 1,178,960 (24%) of the population living in non-metro areas. Rural Alabamians have fewer hospital and clinic resources, with only five rural critical access hospitals, 137 rural health clinics, 120 rural federally qualified health centers, and 49 rural short-term hospitals [14]. Given the limited access to healthcare for rural Alabamians, the county health departments have implemented a project to increase access to healthcare through telehealth services which is now available through 65 of the 67 counties in Alabama [15].

### 1.4. Desired State

The American Academy of Sleep Medicine (AASM) practice guidelines recommend treating OSA with PAP therapy based on a diagnosis of OSA established using objective sleep testing. Good practice recommendations include troubleshooting and monitoring of objective efficacy and usage data to ensure adequate treatment and compliance following PAP therapy initiation and during treatment of OSA. Recently updated practice recommendations suggested behavioral and/or troubleshooting interventions be given during the initial period of PAP therapy in adults with OSA. The AASM also recommends clinicians use telemonitoring-guided interventions during the initial period of PAP therapy in adults with OSA 16. Therapy compliance in the first month of PAP therapy is a long-term predictor of future therapy compliance. According to CMS, the minimal goal for therapy compliance is to wear PAP a minimum of four hours per night for 70% of the time within a consecutive 30-day period within the first 90 days of having the PAP machine [3].

Considering the percentage of rural patients newly diagnosed with OSA who have barriers to care and the recommendations by the AASM, utilizing weekly telehealth visits during the crucial first month of PAP therapy has the potential to significantly improve not only the PAP compliance percentage of rural patients diagnosed with OSA, but their overall health and well-being.

### 1.5. Review of Literature

A literature review utilized key words including obstructive sleep apnea (OSA), rural setting, positive airway pressure (PAP), telehealth, telehealth, and compliance. The search identified 264 articles, which was limited to 121 articles which met the inclusion criteria of being written in English, peer-reviewed, available in full text, and published within the past five years. After reviewing the articles, eight articles with the inclusion of both qualitative and quantitative data were considered relevant.

### 1.6. Journal Article Data Extraction

The articles were reviewed and compared, and the data were organized under the following headings: year, study purpose, study design, main study findings, and conclusion. Although limited to date, emerging research documents the importance of screening for and treating OSA, especially in the rural, underserved populations in the United States. Approximately 19% of America’s population lives in rural areas, which includes 97% of America’s total landmass [16,17]. The eight articles reviewed in this proposal demonstrate the need for screening and treatment of OSA in this rural population, as well as pathways to improve access to care and compliance with prescribed therapies.

### 1.7. Journal Article Data Synthesis

Feltner et al. [18] found that with a diagnosis of OSA, the Epworth Sleepiness Scale (ESS) was beneficial in assessing residual sleepiness during treatment. Labarca et al. [19] also used the ESS to evaluate sleepiness prior to and during prescribed therapy with positive airway pressure (PAP). Lugo et al. [20] used the ESS and Quebec Sleep Questionnaire (QSQ) to assess sleepiness pre-treatment and at various stages during treatment of suspected and diagnosed OSA through both hospital-based and telehealth-based monitoring. The ESS and QSQ provide a universal and consistent standard for subjective documentation of sleepiness.

An elevated body mass index (BMI) is associated with increased risk of OSA. The prevalence of obesity in rural populations is 6.2 times higher than that of urban Americans. Obesity is a leading cause of death in America. Over 60 million obese Americans live in rural areas with lower incomes and less access to medical care than people living in urban areas [17]. Apnea hypopnea index (AHI), blood pressure, and BMI were measured at baseline and throughout treatment of OSA in the systematic reviews of Feltner et al. [18] and Labarca et al. [19].

Labarca et al. [19] evaluated various telehealth strategies to improve compliance with PAP therapy for patients diagnosed with OSA. The meta-analysis found that PAP compliance increased with telehealth visits by 29.2 min each night (*p* < 0.01). Rattray et al. [12] performed a controlled trial with fifty-two patients enrolled through the Veterans Affairs Medical Center who were diagnosed with OSA and managed through a TeleSleep clinic, revealing a cost-effective alternative to traditional hospital-based management. The trial also documented PAP compliance at 180 days to be similar to traditional management (*p*-value = 0.770) and defined disease control as an AHI < 5, which was also similar to traditional management (*p*-value = 0.470). Lugo et al. [20] conducted a randomized control trial in which 186 patients in a Spanish sleep unit were randomized between a telehealth based virtual sleep unit (VSU) and hospital routine management. Again, the VSU was found to be a more cost-effective method of diagnosing and managing OSA than the hospital routine management. Visit time and quality of life measures were significant factors in patient satisfaction with telehealth services.

The evidence report prepared by Feltner et al. [18] documented improved quality of life, sleep-related quality of life, and general health for patients treating OSA with PAP therapy. Lugo et al. [20] utilized the EuroQol (EQ-VAS) to determine that the VSU was more effective in improving quality of life years and overall quality of life as compared with hospital routine management (*p* = 0.046). Rattray et al. [12] found that high patient satisfaction with the TeleSleep program was related to the remote monitoring visits being 50% shorter than in-person visits and saving the patients a mean of seventy-two miles of travel. Most of the study participants lived in rural or very rural areas. This resulted in $29.82 savings for each in-person visit that was avoided. Corrigan et al. [9] also discussed barriers to care, documenting that patients who lived farther from specialist services were less likely to receive timely diagnosis and treatment of OSA. Murphie et al. [11] found potential for telehealth to offer more convenient care and reduce travel, therefore removing barriers to care.

Hu et al. [10] reviewed eleven randomized controlled trials (*n* = 1358) with inclusion criteria that included trials comparing patients who received telehealth interventions with a control group and reported change in PAP compliance. Compared to controls, the telehealth group exhibited better compliance with PAP therapy (pooled mean difference (MD) = 0.57, 95% CI =0.33 to 0.80, *p* < 0.00001). Patil et al. [17] were commissioned by the AASM as a task force to establish practice guidelines for the treatment of adult OSA with PAP. After a systematic review, they recommended that behavioral and troubleshooting interventions be given during the initial period of PAP therapy in adults with OSA. They also suggested clinicians use telemonitoring-guided interventions during the initial period of PAP therapy in adults with OSA.

Many studies assessing the efficacy of telehealth are limited by the number of participants and isolated populations not being reflective of patients living in rural United States. The rural or urban demographic was only evaluated in two of these studies. This information is crucial given the impact on education, resources, and access to care. The TeleSleep program through the Veterans Affairs Medical Center enrolled fifty-two patients in an isolated geographic area and patient population, limiting the study by number of enrollees, geographic location, and population [12]. This pilot program suggested that future efforts may address how to integrate traditional and remote sleep medicine within a healthcare system that serves community-based, non-veteran populations.

### 1.8. Evidence-Based Practice: Verification of Chosen Option

As evidenced by the review of the literature, there is growing evidence to support telehealth in improving PAP compliance in patients with OSA and to remove barriers to care. At the least, telehealth capabilities can be leveraged to provide equal or near-equal PAP therapy initiation and troubleshooting for rural patients in comparison to urban patients with better access to care. Telehealth visits in the initial period of PAP therapy have proven to be effective in improving PAP compliance and are recommended by the AASM. The negative impact that undiagnosed and untreated OSA has on the United States healthcare system and economy is undeniable. Implementing an evidence-based practice of weekly telehealth visits in rural patients diagnosed with OSA during the initial period of PAP therapy has the potential to improve therapy compliance, remove barriers to care, reduce mortality, and improve quality of life for this population.

### 1.9. PICOT

In rural patients diagnosed with obstructive sleep apnea (OSA), do weekly telehealth visits during the first month of positive airway pressure (PAP) therapy improve therapy compliance compared to rural patients who do not receive the telehealth intervention?

### 1.10. Theoretical Framework or Evidence-Based Practice Model

Conceptual frameworks are broad and descriptive. They use high-level processes to move from discovery to action using translation of evidence-based programs, practices, or policies. A conceptual framework is used to illustrate the variables to be studied and the relationships that are expected to be found between them. Knowledge translation has been defined as a process that includes the synthesis, dissemination, exchange, and ethically sound application of knowledge to improve health. Using conceptual frameworks to facilitate knowledge translation provides more effective health services and products and strengthens the healthcare system [21]. Graham’s Knowledge-to-Action (KTA) Framework was developed in 2006 by Dr. Ian Graham and colleagues and is based on over thirty change theories [22]. This framework has demonstrated success in the implementation of both small and large projects. The KTA framework begins with knowledge creation and includes the production, synthesis, and interpretation of knowledge. The action cycle of the KTA framework has seven stages which must all be addressed to produce implementation of knowledge and sustained change.

### 1.11. Knowledge Creation

A knowledge inquiry regarding the lack of resources to screen and treat OSA in rural settings was satisfied during the primary studies (e.g., randomized controlled trials) surrounding this issue. Knowledge synthesis occurred through the collection and interpretation of results from primary studies (e.g., systematic reviews and meta-analyses). Knowledge tools/products (e.g., clinical practice guidelines, decision aids) refer to documents that present synthesized knowledge in a user-friendly format to help health professionals and patients make evidence-based decisions [23]. The research supporting the gap between research findings and clinical practice has led to the clinical guidelines and recommendations found through the National Sleep Foundation [24], AASM and Healthy People 2030. Knowledge becomes more user-friendly as it moves through the three stages of knowledge creation.

### 1.12. Action Cycle

Phase one of the action cycle identifies a problem and determines a gap that ought to be closed. The importance of screening and treating OSA is well documented. Rural populations often lack the resources to implement these measures. SHOP serves to close the gap between research findings and standards of care. Phase two adapts knowledge to the local context by taking into consideration the population and available resources. Implementation of weekly telehealth visits during the first month of PAP therapy adapted SHOP to the targeted population. Phase three of the action cycle assesses barriers/facilitators to knowledge. The multidisciplinary approach included nurses, a physician, and sleep medicine professionals to tailor education and treatment plans for each patient based on resources, educational strengths, and weaknesses. Phase four selects, tailors, and implements patient-specific interventions and occurred during the SHOP screening process based on receptiveness to telehealth visits, internet access, phone access, and need for assistance from caregivers with the visits. Insurance coverage and financial issues impacting the patient’s ability to access PAP machines and supplies were also addressed during this phase. Phase five monitors knowledge use. The weekly telehealth visits assessed patient progress and compliance with therapy. The telehealth visits offered opportunities for patients to ask questions and for the sleep team to adjust therapy based on patient feedback. Remote monitoring of the patient’s therapy compliance occurred throughout this phase. Phase six evaluates outcomes and determines whether the KTA intervention is positively influencing the desired outcomes. The comparison between PAP compliance and ESS scores in the telehealth group and the group that did not receive the telehealth intervention were compared during this phase. Phase seven sustains knowledge use and maintains interventions to sustain the desired outcomes. The telehealth intervention proved beneficial and will be implemented consistently in practice for all patients newly diagnosed with OSA who reside in rural areas [25].

### 1.13. Goals, Objectives, and Expected Outcomes

The goal of this quality improvement (QI) project was to improve PAP therapy compliance in rural patients diagnosed with OSA by removing barriers to care. The objective of the principal investigator (PI) was to identify a group of rural patients newly diagnosed with OSA and conduct weekly telehealth visits during their first month of PAP therapy. Implementation of telehealth provided support and troubleshooting during this crucial period. Compliance with PAP therapy was monitored remotely by downloading the machine usage data. The Epworth Sleepiness Scale (ESS) was used to assess reported sleepiness prior to and during PAP therapy. The efficacy of the telehealth intervention was determined by comparing PAP compliance data and ESS scores to a group of rural patients who did not receive the telehealth intervention. The project improved both PAP compliance percentages and ESS scores in the telehealth group.

### 1.14. Facilitators

Setting facilitators included a large rural referral base to the practice and a rural clinic site which houses a credentialed sleep center. Another setting facilitator was the electronic health record used by the practice, which is easily accessible and can extract data based on identifiers such as diagnosis and rural zip codes. The ability to access machine downloads for review and treatment adjustments also provided an environment for success of the project. Utilizing telehealth visits was considered a facilitator, as these visits were conducted without travel or expense for the patient. These visits were conducted by both phone and video based on patients’ access to a smartphone and internet connection. Video visits were performed through a secure application already used within the practice. The first month of PAP therapy is crucial. Since the AASM recommends telemonitoring and troubleshooting during the initial period of PAP therapy, this project implemented the highest evidence-based practice guidelines [3].

### 1.15. Barriers

Since people living in rural areas have limited access to healthcare services and numerous barriers to care, the rural population of this project was a potential barrier. There are several social determinants that are barriers for rural communities. Extreme poverty rates can make it difficult for participants to pay for services or programs. Cultural and social norms surrounding health behaviors, low health literacy levels, and incomplete perceptions of health in rural communities can be barriers to health promotion. Limited affordable, dependable, or public transportation combined with unpredictable work hours or unemployment also pose challenges for these communities [14]. The project design of utilizing telehealth minimized some of these barriers.

Practice barriers included an increased time burden on the PI and limited hours during the day to perform the telehealth visits. In the current office practice, patients are often difficult to reach at the scheduled appointment times and may not prioritize the visits as they do in-person visits. This issue necessitates numerous attempts to reach patients and increases the burden on office staff, increasing the possibility of resistance from practice administration. The time constraint of four weeks limits the number of participants who were enrolled in the QI project.

## 2. Materials and Methods

### 2.1. The Pilot Study

**Figure 1:**
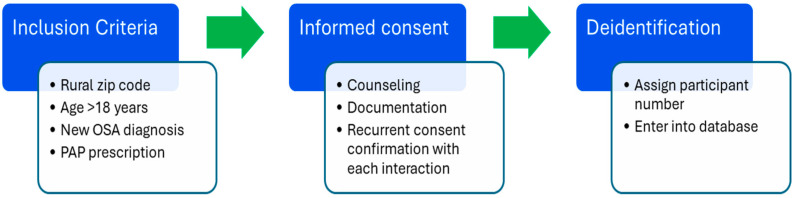
Program Entry Phase. The flow diagram tracks the participant enrollment process.

As SHOP was developed, eligible patients were enrolled in the pilot study. Informed consent was obtained from the participants, and they were assigned a participant number (Figure 1). The patient’s personal health information was not attached to the participant number. An Excel spreadsheet was developed and shared with team members to record enrollment, informed consent, telehealth visits, Likert scale scores from weekly and exit questionnaires, Epworth Sleepiness Scale (ESS) scores, and positive airway pressure (PAP) machine compliance data. A data dictionary was available to team members which defined the definition of PAP compliance as using the PAP system at least four hours a night for 70% of the time over one 30-day period in the first 90 days of therapy. This guideline for compliance is also enforced by some insurance carriers and serves as a general guideline for therapeutic PAP usage. Weekly telehealth visits were performed for the first four weeks of PAP therapy using a questionnaire as a guideline for the visit. The weekly visits provided an opportunity for troubleshooting and therapy adjustments. Simultaneous to the study, retrospective were was obtained from the practice EHR for compliance data during the first four weeks of PAP usage for patients residing in rural zip codes who did not receive the telehealth intervention.

The therapy compliance percentage not only indicates whether some patients meet insurance requirements to keep the PAP machine but indicates successful treatment of the OSA and potential improvement in health outcomes. PAP machine downloads were obtained using the machine modem or SD cards. The results were easily summarized and analyzed as the compliance percentage of patients without the telehealth intervention was compared with the compliance percentage of patients with the telehealth intervention. The benefit of telehealth was apparent since the compliance percentages and usage days were higher with the telehealth intervention.

### 2.2. Project Design

This quality improvement (QI) pilot study implemented weekly telehealth visits during the first four weeks of PAP therapy to improve PAP compliance in rural patients diagnosed with OSA. The compliance data were compared with rural patients who did not receive the telehealth intervention. Both quantitative and qualitative data were obtained. The quantitative data included machine download data and compliance percentages from the EHR and cloud database for the machines. Qualitative data were obtained from both the weekly questionnaire and exit survey for participants regarding their opinions and acceptance of telehealth interventions about improving access to care and therapy compliance. Data types monitored in the program include administrative, patient-reported, and PAP system data. Administrative data include enrollment, consent, and telehealth schedule information. Patient-reported data include Likert scales, sleep questionnaires, and the Epworth Sleepiness Scale. PAP system data include compliance download information and leak data.

### 2.3. Project Site and Population

The project site was a neurology and sleep medicine practice with a primary outpatient clinic in Tuscaloosa, Alabama, and a remote clinic site in rural Demopolis, Alabama. The practice serves West Alabama communities, although the patient population represents patients from all fifty states of the United States. Tuscaloosa County is 74% urban and 26% rural, with a population of approximately 236,780 [26]. Demopolis, Alabama is in Marengo County and has an estimated population of 19,323 [27]. There are sleep lab sites in both Tuscaloosa and Demopolis, and both participated in the study. The population for the study included patients newly diagnosed with OSA and prescribed PAP therapy who reside in rural zip codes. Retrospectively, the control group of patients included patients who were diagnosed with OSA, prescribed PAP therapy, and did not receive the telehealth intervention. The goal of enrollment in the study was thirty patients in the telehealth intervention group and thirty patients in the control group. Twenty-six patients were enrolled in the study, but two patients did not complete the study, resulting in twenty-four patients in the intervention group and twenty-four patients in the control group. The resources required for this project were minimal and included the PI’s time as the provider and collaboration and time with the sleep program coordinator to identify eligible participants and obtain PAP machine data downloads. The clinic EHR was used for secure record keeping and running reports to identify patients by zip code.

### 2.4. Measurement Instruments

The outcomes of this project were measured with PAP compliance percentages, ESS scores, weekly visit questionnaires, and an exit interview questionnaire. The PAP compliance percentage is automatically calculated by the manufacturer’s remote application based on the date of machine set-up, hours of usage, and end of the designated monitoring period. The instrument is easy to use and access remotely. Missing data would be due to no machine usage or a lack of cellular service and modem capability. With no machine usage, the compliance is documented as 0% and the patient is documented to be non-compliant with the therapy. The SD card in the PAP machine can be downloaded and reviewed in cases complicated by poor cellular service or the lack of a modem.

The ESS (Appendix A) is the tool that was used to assess daytime sleepiness initially and while using PAP therapy. The ESS was developed by Dr. Murray Johns in 1990 and modified in 1997. The tool is protected by copyright and requires a license to use, although a license fee is not payable by students, physicians, clinical practices, and not-funded academic users. The licenses and ESS document download can be obtained through the ESS website, which is managed through the Mapi Research Trust [28].

The ESS contains eight questions which allow respondents to rate their usual chance of dozing off or falling asleep while engaged in eight different activities using a four-point scale. The higher the ESS score, the higher the respondent’s average sleep propensity in daily life. The tool takes 2–3 min to complete and is available in many different languages. ESS is a unitary scale that is reliable and valid for measuring daytime sleepiness. The tool is cheap and easy to use for individuals and large groups. Scores range from 0–24 and the reference range of ‘normal’ ESS scores is zero to 10 [29]. The psychometric properties of the ESS have been investigated widely. The internal consistency has been tested in ten separate investigations using Cronbach’s alpha, which varied between 0.73 and 0.90 (mean 0.82). The test-retest reliability of the ESS scores (measured over a few weeks to a few months) has been tested by the intraclass correlation coefficient, which varied between 0.81 and 0.93 in five separate investigations [29].

Likert scales are a cornerstone of satisfaction measurement, offering a straightforward, powerful method for quantifying subjective experiences. Likert scales present respondents with a series of questions, prompting them to indicate their level of satisfaction along a symmetrical, ordered scale. In healthcare, they can measure patient satisfaction with treatments and access to care. The ease of use for respondents and the generation of quantifiable data for statistical analysis are key advantages. Likert scales provide a range of responses that progress in a logical order, from one extreme to the other (e.g., “very dissatisfied” to “very satisfied”).

The weekly telehealth visits were guided by 5-point Likert questionnaires assessing how well patients are tolerating PAP therapy, how well they are tolerating the mask, and their sleep quality using the PAP (Appendix B). Likert questionnaires also investigated satisfaction with care and satisfaction with access to care. An exit interview utilized a three-question 3-point Likert questionnaire to assess improvement in sleep quality related to PAP therapy, whether they believe telehealth has been beneficial in their adjustment to PAP therapy, and whether they feel telehealth has improved their access to healthcare (Appendix C).

### 2.5. Data Collection Procedures

**Figure 2:**
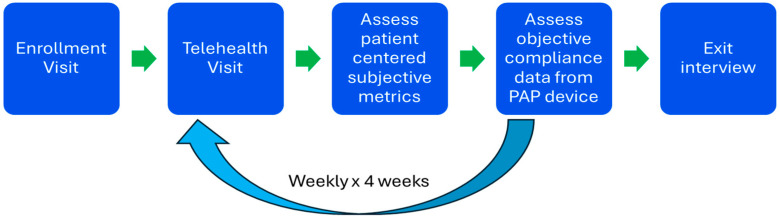
Program Monitoring Phase. After enrollment, the patient participates in a telehealth visit with assessment of subjective patient-centered metrics and objective device metrics in a recurring weekly protocol for 4 weeks.

Data collection began with enrollment and an initial ESS score of each participant. The PAP compliance data and weekly telehealth questionnaire scores were obtained each week for four weeks (Figure 2). The investigator contacted the participants for the weekly telehealth visits via the telehealth system utilized by the clinic for standard telehealth visits. This system utilizes the Doximity platform, which is a secure HIPAA-compliant telehealth system. Each telehealth visit lasted an average of 10 min. The topics covered in each visit included system use, PAP tolerance, problems encountered, methodologies for addressing any problems, and the questionnaires and Likert scales as described. The exit interview questionnaire, final ESS score, and final PAP compliance percentage completed data collection. The PI performed the telehealth visits and recorded the data on an Excel spreadsheet, which was protected and stored securely in the practice site EHR and secure server.

### 2.6. Data Analysis

To assess the benefit of the telehealth intervention, compliance data were compared between the telehealth group and the group that did not receive the telehealth intervention. The t statistic (t-test) was used to determine whether there was a statistically significant difference in the mean values between the two groups. The t-test is an appropriate measure for evaluation of these quantitative data as it is often used in hypothesis testing to determine whether a treatment or process influences the population of interest or whether two groups are different from one another [30]. The ESS scores of both groups were compared in the same manner. The Likert scale data from the weekly questionnaires and exit interview questionnaires were evaluated using the descriptive statistics of mean and median [31].

### 2.7. Ethical Considerations/Protection of Human Subjects

Approval was obtained from the University of Alabama (UA) Institutional Review Borad (IRB) prior to initiating the project. The official IRB protocol was prepared as soon as the proposal was approved. All participants were protected by the Health Insurance Portability and Accountability Act of 1996 (HIPPA) which, among other guarantees, protects the privacy of patient’s health information (Modifications to the HIPPA Privacy, Security, Enforcement, and Breach Notification Rules, 2013). Additionally, Standards of Care for practice in a sleep medicine office were carefully followed. After the explanation of the program, the investigator confirmed the patients’ understanding and comfort with telehealth versus traditional in-person follow-ups. All information collected as part of evaluating the impact of this project was aggregated from the project participants and did not include any potential identifiers.

The risk to patients participating in this project was no different from the risks of patients receiving standard sleep medicine care. Participant confidentiality was assured by coding the participants using unique identification numbers. The list of participants and their identifying numbers was kept in a locked filing cabinet in the practice office, only accessible to the principal investigator. All electronic files containing identifiable information were stored on the HIPPA secure UA Box.

## 3. Results

Beginning on 10 January 2024, all patients newly diagnosed with OSA who reside in rural zip codes were assessed for inclusion in the Sleep Health Optimization Program. A total of twenty-six patients were found to meet inclusion criteria and enrolled in the DNP quality improvement project. These patients were all over 18 years of age, lived in rural zip codes, diagnosed with OSA, prescribed PAP therapy, and received a PAP machine. The population consisted of sixteen females and ten males. Two did not complete the project, leaving a total of 24 patients in the intervention group (*n* = 24). The ESS and compliance percentages of the intervention group were compared with those of the twenty-four rural patients newly prescribed PAP therapy who did not receive the telehealth intervention.

When reviewing the use of the telehealth intervention during the first four weeks of PAP therapy, an independent samples t-test was used to compare therapy compliance at 30 days for the intervention versus control groups. Data analysis revealed the intervention group used the PAP machine 79.8% of the time during the first 30 days, whereas the control group used the PAP machine 61.2% of the time during the first 30 days of having the machine. Compliance percentages were higher in the intervention group (63.2%) as compared to the control group (46.4%). There was no significant difference in compliance for the intervention group (M = 63.22, SD = 32.78) versus the control group (M = 46.40, SD = 36.24), t (46) = 1.69, *p* = 0.099. A paired-samples t-test was used to compare ESS scores prior to and after one month of therapy. This analysis revealed that ESS scores were significantly greater prior to one month of therapy (M = 8.38, SD = 5.70) compared to after one month of therapy (M = 2.83, SD = 2.65), t (23) = 5.22, *p* < 0.001, d = 1.07. The exit interview feedback from all patients revealed that participants felt the telehealth intervention was beneficial in their adjustment to PAP therapy (100%) and improved their access to healthcare (100%). The study results are summarized in Table 1.

One patient (#9) in the protocol had no usage. This individual had no change in the ESS, which is to be expected since the patient was not compliant with therapy. Another patient (#14) had relatively low compliance but was utilizing the system. There was a significant improvement in the ESS for this individual. This could be secondary to actual improvement from partial use or possibly an effect created by the frequent contact by the research team.

In addition to the initial pilot program assessment, the participants in the pilot program were compared to the compliance and satisfaction of patients from rural areas managed according to the standard office-based protocol being used. Information obtained from the durable medical equipment companies revealed that patients managed by this practice with standard approaches to care had a compliance rate of 86% for all patients in the practice. The compliance rate for patients managed by the same protocols but residing in rural areas was 58%.

## 4. Discussion

The goal of this quality improvement project was to improve access to healthcare and improve PAP compliance during the first 30 days of therapy for patients living in rural areas. Telehealth visits resulted in a trend toward improved PAP therapy compliance in the intervention group, and although not statistically significant at 30 days, the PAP usage percentage was also higher in the intervention group. Since PAP usage in the first month of therapy is a long-term predictor of PAP compliance, the intervention was felt to be beneficial. National averages for PAP compliance typically range from 30% to 60%.

There are several factors contributing to the *p*-value of 0.099. The two most important factors are the small sample size utilized in this pilot trial and the relatively short duration of intervention. Importantly, one measure of success for this pilot study is not superiority over traditional approaches but equivalent compliance rate success without the need for travel for this rural population.

The overall compliance rate, and more specifically the compliance rate for urban or suburban patients seen in the clinic, is superior to that of patients residing in rural areas. This significant reduction represents the effect of the barriers to care faced by this population. The utilization of the enhanced SHOP telehealth approach in the rural population of patients resulted in a compliance rate that was better than the results of standard care for a similar population. The overall population was not assessed in the highly standardized fashion utilized in this study. This creates a limitation of the study which will be further evaluated in a streamlined and optimized version of this protocol to be utilized in a larger formal outcomes trial.

The patients in the study were extremely pleased with the quality of care, access to care, and ease of follow-up as assessed by a Likert scale questionnaire. All participants rated these metrics as “5/5” or “extremely satisfied”. A recent study on the effects of telemonitoring on PAP adherence did not reveal any significant improvement in adherence, but this study did not assess patient satisfaction in detail [32].

In comparison, review of the comparison group of standard-approach patients revealed “5/5” for quality of care, but “4.5/5” for access to care and “4.2/5” for ease of follow-up. Similarly, the Likert scores for the overall clinic population being treated with standard approach patients revealed “5/5” for quality of care, but “4.5/5” for access to care and “4.1/5” for ease of follow-up. The Likert scores for the rural population being treated with standard-approach patients revealed “5/5” for quality of care, but “4.0/5” for access to care and “3.8/5” for ease of follow-up—much lower than the results for either the study group or the general clinic population.

The correlation between PAP adherence and the quality of the physician–patient relationship is undeniable, serving as a critical factor in successful OSA management. Effective communication forms the bedrock of this connection. Scheduled and frequent monitoring and follow-up appointments reinforce commitment to the patient’s well-being, providing opportunities to address challenges and adjust treatment as needed. Ultimately, a strong physician–patient relationship translates to increased trust, enhancing the patient’s willingness to adhere to PAP therapy and thus improving their overall health outcomes.

Telehealth follow-up for compliance enhancement is cost-effective for both the practice and the patient. The significant barriers to care for the patient, including travel cost and inconvenience, are minimized with this approach. The no-show and cancellation rates decrease with this approach as well, improving practice productivity. The significant improvement in Likert scores for “access to care” and “ease of follow-up” support use of this approach.

The limitations of this study include that it was initiated in January—the height of cold and flu season—and that many participants delayed initiation of PAP therapy or had limited usage at times related to cough and congestion. This was a small pilot study and was compared, in part, to a larger general population sample. Frequent telehealth contact can create an effect of improved responses to subjective questioning. The lack of blinding for both participants and investigators introduces potential bias in reported outcomes, particularly for subjective measures. This issue is common in telehealth studies and can impact data reliability. A longer tern follow-up evaluation period would enhance the evaluation of this program. A program between 3 and 6 months is planned. This will be optimized by utilizing the lessons learned during this pilot study.

This study focuses on the population in the Black Belt region of Alabama, which is one of the most underserved areas of the country. Residents of these counties face numerous potential barriers to care because of rurality, poverty, limited access, and educational limitations, among others. The approach to managing the barriers to care in this region has been used in the development of multiple state and federal programs, displaying the generalizability of an interventional study in this population. Given the barriers to care in this population, a successful program in this group is likely to be successful in other rural and underserved populations. This approach can also serve as a framework for programs in populations not faced with these challenges.

The telehealth visits provided an opportunity to help patients adjust their humidification and mask options to accommodate for these issues. Since therapy compliance numbers continued to improve over the thirty-day study period, recommendations for future research would include extending the monitoring period to reflect the full benefit of the telehealth intervention.

## 5. Conclusions

The negative impact that undiagnosed and untreated OSA has on the United States healthcare system and economy is undeniable. Compliance with PAP therapy for patients diagnosed with OSA is key to improving the symptoms associated with untreated OSA and reducing comorbidities. Aligning with the goals of Healthy People 2030 and the evidence-based practice guidelines established by the AASM, this pilot study implemented weekly telehealth visits during the crucial first month of PAP therapy to remove barriers to care and improve PAP therapy compliance in the rural patients enrolled in this pilot study. The telehealth intervention implemented in this pilot study improved access to healthcare for rural patients, yielded a trend toward improved PAP therapy compliance, and revealed a high level of patient satisfaction with the telehealth interventions.

## Figures and Tables

**Table 1:** Participant results of the pilot study group.

Participant Number	Inital ESS	Exit ESS	Compliance % at 30 Days	Usage Days %
1	3	1	93.3	100
2	2	2	93.3	100
3	16	1	83.3	83.3
4	5	3	100	100
5	21	2	43.3	100
6	11	3	100	100
7	5	2	50	57
8	4	1	66.7	90
9	12	12	0	16.7
10	8	3	33.3	93.3
11	4	3	93.3	96.7
12	0	0	7	10
13	11	3	63	97
14	22	7	50	70
15	4	4	60	87
16	3	2	27	57
17	14	6	96.7	96.7
18	10	4	63	67
19	7	0	97	100
20	8	0	40	73
21	11	3	0	33.3
22	5	0	100	100
23	10	2	87	100
24	5	4	70	87
	8.4	2.8	63.2	79.8

**Figure A1:**
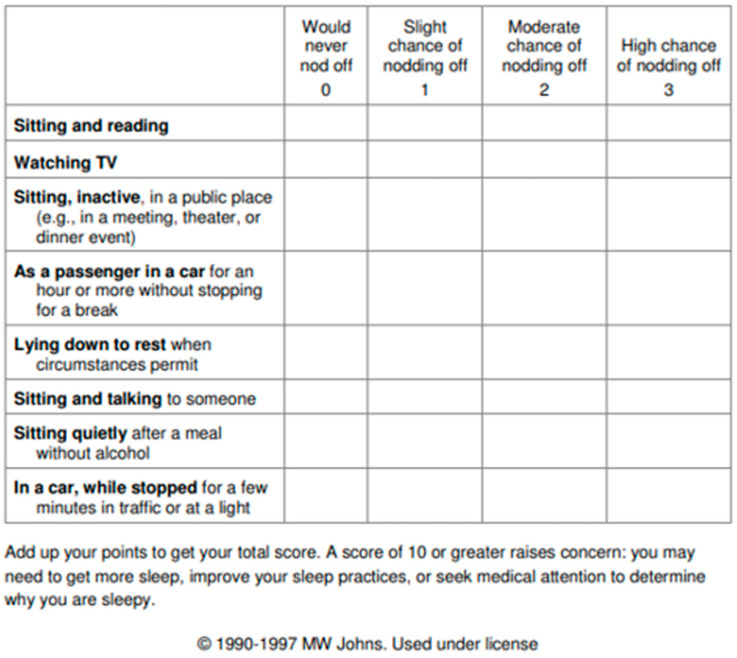
Epworth Sleepiness Scale form.

## Data Availability

Data availability status Recommended Data Availability Statement. Data are contained within the article. The original contributions presented in the study are included in the article, and further inquiries can be directed to the corresponding author/s. The dataset will be made available by the authors on request. The raw data supporting the conclusions of this article will be made available by the authors on request.

## Appendix A. Epworth Sleepiness Scale (ESS)

How likely are you to doze off or fall asleep in the following situations in contrast to feeling just tired? This refers to your usual way of life in recent times. Even if you have not done some of these things recently, try to work out how they would have affected you. It is important that you answer each question as best you can. Use the following scale to choose the most appropriate number for each situation.

## Appendix B. Weekly Telemedicine Visit Questionnaire

**The responses will be rated on the following 5-point Likert scale**				
1-not at all				
2-some good nights, but mostly challenging nights				
3-as to be expected, adjusting				
4-more good nights than bad nights				
5-very well				
**1. How well are you tolerating your positive airway pressure (PAP) therapy?**				
1	2	3	4	5
**2. How well are you tolerating your mask?**				
1	2	3	4	5
**3. How is your sleep quality using the PAP?**				
1	2	3	4	5
Notes: Primary investigator to document total number of hours the patient is using the PAP and how many total hours they are sleeping. Also, the need for therapy adjustments will be documented.				

## Appendix C. Exit Questionnaire

**The responses will be rated on the following 3-point Likert scale**		
1-not at all		
2-undecided		
3-definite benefit		
**1. Do you feel the PAP therapy has improved your sleep quality?**		
1	2	3
**2. Do you feel the telemedicine intervention has been beneficial in your adjustment to PAP therapy?**		
1	2	3
**3. Do you feel telemedicine has improved your access to healthcare?**		
1	2	3
